# Impact of Body Mass Index on the Outcomes of Intensive Complex Decongestive Therapy for Lower Limb Lymphedema

**DOI:** 10.1155/ijvm/8951146

**Published:** 2026-01-27

**Authors:** Marie Warnier, Nina Antoniolli, Benoit Bihin, Jacqueline Frippiat, Chloé Meseeuw, Alexis Lheureux, Thierry Deltombe

**Affiliations:** ^1^ Department of Physical Medicine and Rehabilitation, CHU UCL Namur-Godinne Site, Yvoir, Belgium

**Keywords:** body mass index, complex decongestive therapy, functioning, lymphedema, volume

## Abstract

**Background:**

Complex decongestive therapy is recognized as the primary treatment of lymphedema. While the influence of weight on the lymphedema appearance and evolution is well known, the relationship between weight and complex decongestive therapy outcome remains underexplored.

**Purpose:**

The purpose of this research is to evaluate the impact of body mass index on the effectiveness of intensive complex decongestive therapy in patients with primary and secondary lower limb lymphedema.

**Methods:**

We conducted a prospective study on 159 patients who underwent 551 complex decongestive therapy programs at the CHU UCL Lymphedema Reference Center from April 1, 2018, to March 19, 2021. Patients were categorized by body mass index ranges (18.5–24.9, 25–29.9, 30–34.9, 35–39.9, and 40–55 kg/m^2^). Before and after treatment, limb volumes were calculated using the truncated cone formula and functional impairments using the Lymph‐ICF‐Lower‐Limb questionnaire. Linear regression models were used to evaluate the association between weight categories and treatment outcomes and were adjusted for relevant confounding factors.

**Results:**

Higher body mass index was associated with larger initial lymphedema volumes and lower functional status. Complex decongestive therapy reduced the relative lymphedema volume of 7% and improved functional status irrespective of body mass index groups.

**Conclusion:**

Although weight is correlated with an initial greater lymphedema volume and a lower functional status, it does not influence the ability of complex decongestive therapy to reduce limb volume and improve function. Complex decongestive therapy is recommended regardless of a patient′s body weight.

## 1. Introduction

The lymphatic system plays a crucial role in fluid balance and immune function by transporting lymph through vessels and lymph nodes into the bloodstream [1]. When this system malfunctions, lymphedema can develop, characterized by protein‐rich fluid accumulation in the interstitial space, leading to swelling. This swelling can lead to increased adipose tissue deposition and cutaneous fibrosis over time. Lymphedema commonly affects the upper and/or lower limbs (LLs) but can also involve the genitals, face, neck, chest wall, and oral cavity [2, 3].

Lymphedema is classified into primary, resulting from congenital lymphatic system abnormalities [4], and secondary, often occurring after cancer treatments [5], infections, trauma [6], or rheumatological conditions. Primary lymphedema affects approximately 1.15 per 100,000 people under the age of 20 [7], but its prevalence is difficult to estimate due to challenges in diagnosis. The prevalence of secondary lymphedema after lymph node resection is notably high; approximately 30% of breast cancer patients and 20% of melanoma patients are affected [8]. Diagnosis typically relies on clinical evaluation, including features such as pitting edema and a positive Stemmer′s sign [9–12], with lymphoscintigraphy used for confirmation [13].

Lymphedema is classified into five stages based on severity, ranging from subclinical stasis (Stage 0) to severe lymphostatic elephantiasis (Stage 3) [14]. Lymphedema volume is measured by means of the volume water displacement method and/or by the circumferential measurement and volume calculation using the truncated cone formula [15]. The difference between limbs is assessed by the percentage of excess volume (PEV) compared to the healthy limb, in cases of unilateral lymphedema. A difference of less than 10% is generally not indicative of lymphedema [16–20]. However, some studies suggest that a percentage as low as 5% [21] or even 3% [22] may also be relevant. The International Society of Lymphology defines mild (5%–19% PEV), moderate (20%–40%), and severe (> 40%) lymphedema [14]. In cases of bilateral lymphedema, which are more frequently observed in the LLs, PEV is not a reliable metric for assessing severity.

The cornerstone of lymphedema management is the complex decongestive therapy (CDT), which comprises an intensive phase aimed at reducing edema, followed by a maintenance phase to sustain the improvements [22]. CDT includes compression therapy using short‐stretch bandages, exercise, pneumatic compression, manual lymphatic drainage (MLD), and skin care. Long‐term management involves the use of compression garments and exercises [5, 23]. The impact of lymphedema on functioning and quality of life is assessed by the Lymph‐ICF questionnaire, available for upper (Lymph‐ICF‐UL) and lower (Lymph‐ICF‐LL) limb lymphedema [24–27].

While prior studies have explored the role of body mass index (BMI) in the development of lymphedema [28–30], particularly in cancer‐related cases [31–33], the relationship between BMI and the efficacy of CDT remains underexplored. Some studies have suggested a potential association between higher BMI and reduced therapeutic response, particularly in cancer‐related upper limb lymphedema [34] or in elderly populations [35]. However, these studies either focus on different anatomical sites or lack a dedicated statistical analysis on BMI as a predictive factor. To our knowledge, no study has specifically and systematically evaluated this relationship in LL lymphedema.

This study is aimed at investigating the association between BMI and the outcomes of the intensive phase of CDT in patients with primary and secondary LL lymphedema. The underlying hypothesis is that higher BMI may be associated with less favorable therapeutic outcomes, both in terms of volume reduction and functional improvement.

## 2. Methods

### 2.1. Study Design

This prospective study analyzed clinical data from patients who underwent sessions of CDT for primary and secondary LL lymphedema. The study was conducted at the CHU UCL site Godinne Lymphedema Reference Center (Belgium) between April 1, 2018, and March 19, 2021. Ethical approval was obtained from the Ethics Committee of CHU UCL Namur. The study adhered to the National Institute for Health and Disability Insurance Reference Center Lymphedema Convention and the Declaration of Helsinki (2013 revision). All participants provided written informed consent for the use of anonymized data.

### 2.2. Setting and Participants

Clinical and demographic data were collected through assessments performed by trained clinicians. Inclusion criteria were as follows: age over 18 years, unilateral or bilateral LL lymphedema, either primary or secondary, lymphedema classified as Stage 2, late Stage 2, or Stage 3 according to the 2020 International Society of Lymphology staging system [14], a BMI between 18.5 and 55 kg/m^2^ (weight in kilograms divided by height in meters squared), and participation in a CDT program. Exclusion criteria were upper limb lymphedema, BMI outside the specified range, a limb volume difference below 5% in unilateral cases, CDT administered more than 1 year after the previous treatment, as this delay exceeds the expected duration of the maintenance phase, missing data, or incomplete CDT programs.

A 1‐year interval was selected to reduce variability related to prolonged treatment gaps. This choice was based on prior findings by Ko et al. [36] and Vignes et al. [37], which demonstrated that volume reductions achieved with CDT can be sustained for up to 12 months. These studies support the rationale for using a 1‐year cutoff in our inclusion criteria. BMI limits helped mitigate confounding from nutritional extremes, and the 5% asymmetry threshold in unilateral cases aligned with prior studies identifying this value as a clinically relevant early indicator of progression [38].

All patients received a standardized CDT program consisting of multilayer bandaging, physical exercise (e.g., strengthening, cycling, and Nordic walking), skin care, patient education, pneumatic intermittent compression (PIC), and MLD. Each session lasted 5 h per day over 5 consecutive days.

Therapeutic exercise under compression represented the most important component of the CDT program, as it actively stimulated the muscle pump and enhanced lymphatic drainage [14]. Short‐stretch multilayer bandages were applied daily by certified lymphedema therapists and maintained during all daytime activities. MLD was performed for approximately 30 min per day, and PIC for 30 min using calibrated medical‐grade devices set between 40 and 60 mmHg, in accordance with the International Society of Lymphology consensus guidelines [14]. The remaining time was devoted to skin care and patient education to promote self‐management and maintenance of decongestion. This standardized protocol ensured that all participants received a consistent, intensive CDT regimen.

### 2.3. Outcomes and Measurements

The primary outcome was LL volume, expressed in liters, calculated using the truncated cone formula based on circumferential measurements taken every 4 cm. The formula used was volume = *h* (*C* ^2^ + *C* *c* + *c* ^2^)/(12 × *π*), where *h* is the distance between two measurements (4 cm), *C* is the largest circumference, and *c* is the smallest. Although this formula produces volumes in cubic centimeters (equivalent to milliliters), values were converted to liters to facilitate interpretation, given the typically large limb volumes. The sum of these segment volumes provided the total limb volume. Volume reductions were calculated both as the absolute difference in liters (BT − AT) and as the relative percentage change ((BT − AT)/BT) and also expressed as the log‐ratio of posttreatment to baseline volume (log(AT/BT)).

The secondary outcome was the validated French version of the Lymph‐ICF‐LL self‐questionnaire [39, 40]. This tool evaluates impairments in function, activity limitations, participation restrictions, and quality of life in lymphedema patients, in accordance with the International Classification of Functioning, Disability, and Health (ICF) of the World Health Organization (WHO) [26, 39, 40].

The Lymph‐ICF‐LL questionnaire is a validated tool. The French version used in this study was developed and validated by De Vrieze et al. [40] and is available for noncommercial research use. The full French version of the questionnaire is provided as supporting information (available here). No additional permission was required for its use in this study.

The questionnaire consists of 28 items scored from 1 to 10 or marked “not applicable” (excluded from scoring). The total score was calculated as follows: (sum of item scores ÷ number of items answered) × 10. According to the WHO taxonomy, impairments in function, activity limitations, and participation restrictions can be quantified using the following scale: A score between 0 and 4 indicates no problem; 5–24, a small problem; 25–49, a moderate problem; 50–95, a severe problem; and 96–100, a very severe problem [26, 40, 41]. Scores range from 0 to 100, with higher scores indicating greater impairments.

Changes in specific items were also analyzed, including pain (Item 1), heaviness (Item 6), loss of self‐confidence (Item 7), and difficulty wearing desired clothing (Item 27) to gain insight into the impact of CDT on physical, mental, and social outcomes. These items were selected based on their frequent reporting in clinical practice.

### 2.4. Bias Management

Standardized protocols, validated tools, and uniform data collection by a single trained team minimized bias. Including all eligible patients from the predefined period helped reduce selection bias, and eligibility criteria ensured comparable patient characteristics. Statistical models were used to account for potential confounding factors.

### 2.5. Statistical Methods

All statistical analyses were performed using R Version 4.1.1. Descriptive statistics, including medians and interquartile ranges (IQRs), were calculated to summarize limb volume, Lymph‐ICF‐LL scores, and symptom questionnaire responses across BMI categories (18.5–24.9, 25–29.9, 30–34.9, 35–39.9, and 40–55 kg/m^2^). Wilcoxon signed‐rank tests were used to assess within‐group changes before and after treatment.

To evaluate the impact of CDT, linear regression models were constructed with the log‐transformed ratio of posttreatment to baseline limb volume as the dependent variable. Subgroup analyses were performed based on BMI categories, with the 18.5–24.9 kg/m^2^ group serving as the reference. Additional models explored the effects of BMI on total Lymph‐ICF‐LL scores and on specific functional items (pain, heaviness, self‐confidence, and clothing difficulties). Interaction terms were used to explore subgroup‐specific differences.

Analyses excluded missing data. Sensitivity analyses were conducted to confirm the robustness of the findings.

### 2.6. Use of AI Tools

A generative AI tool (ChatGPT) was used only to translate parts of the manuscript from French to English. All scientific content, data interpretation, and conclusions were produced by the authors, who reviewed and take full responsibility for the final text.

## 3. Results

**Figure 1:**
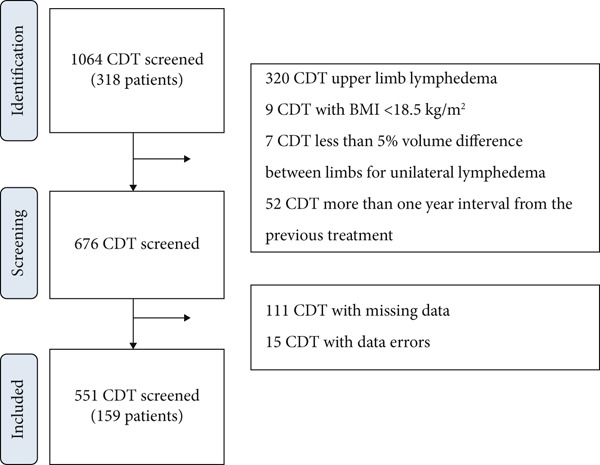
Treatment selection flowchart.

The participants and study flowchart are detailed in Figure 1.

Baseline demographic and clinical characteristics of the study population are summarized in Table 1. Primary lymphedema accounted for the majority of cases (69%). Patients with primary lymphedema were generally younger and more frequently female than those with secondary forms. Bilateral involvement was predominant (64%), mainly among primary cases.

**Table 1:** Baseline demographic and clinical characteristics of the study population (*n* = 159).

	**Total (** **n** = 159**)**	**Primary (** **n** = 110**, 69%)**	**Secondary (** **n** = 49**, 31%)**
Age (years, mean ± SD)	65.0 ± 13.7	64.8 ± 14.1	67.8 ± 12.8
Female, *n* (%)	113 (71%)	81 (73%)	32 (65%)
BMI (kg/m^2^, median [IQR])	27 [26–38]	34 [29–39]	28 [24–35]
Laterality, *n* (%)			
Unilateral	56 (35%)	21 (19%)	35 (71%)
Bilateral	103 (64%)	89 (81%)	14 (29%)

*Note:* The table presents baseline demographic and clinical characteristics of the study population. Values are expressed as mean ± standard deviation (SD) or median [interquartile range, IQR], unless otherwise specified.

A total of 159 patients (71% women, mean age of 65 years) completed 551 CDT sessions. The BMI distribution was as follows: 18–25 kg/m^2^ (*n* = 36), 25–30 kg/m^2^ (*n* = 29), 30–35 kg/m^2^ (*n* = 38), 35–40 kg/m^2^ (*n* = 32), and 40–55 kg/m^2^ (*n* = 24). Most patients had primary lymphedema (69%) and bilateral involvement (67%).

Among the 49 patients with secondary lymphedema (31%), most cases were related to cancer treatment. Gynecologic cancers were the leading cause (*n* = 22, 45%), followed by urologic malignancies (*n* = 13, 27%), melanoma (*n* = 4), soft‐tissue sarcoma (*n* = 3), lymphoma (*n* = 3), a Merkel cell neuroendocrine tumor (*n* = 1), and one benign vascular tumor; one additional patient developed lymphedema after an isolated inguinal lymph node biopsy. Surgical oncologic procedures were almost universal in this cohort, with 41 patients (84%) undergoing lymph node dissection, often in combination with extensive pelvic, inguinal, or abdominal surgery. Radiotherapy was administered in 21 patients (43%), and chemotherapy in 16 patients (33%). Overall, multimodal treatment—including surgery, lymphadenectomy, radiotherapy, and/or chemotherapy—was frequent.

Among the 159 included patients, 75 underwent their first CDT session during the study period. The remaining 476 sessions were follow‐up treatments involving 122 patients; some were included in both categories, depending on their treatment sequence.

All outcome analyses were performed at the session level to evaluate the impact of each CDT intervention independently.

Descriptive statistics and outcomes are summarized in Tables 2, 3, 4, and 5. Table 2 presents baseline and posttreatment limb volumes by BMI category. Table 4 reports baseline and posttreatment Lymph‐ICF‐LL total scores, and Table 5 focuses on four symptom‐specific questionnaire items: pain (Q1), heaviness (Q6), self‐confidence (Q7), and difficulty wearing desired clothing (Q27).

**Table 2:** Limb volume at baseline and posttreatment.

**BMI category (kg/m** ^**2**^ **)**	**Baseline limb volume (BT) (L, median, IQR)**	**Posttreatment limb volume (AT) (L, median, IQR)**
[18–25] *N* = 101	8.9 (7.4, 10.4)	8.33 (7.03, 9.49)
[25–30] *N* = 116	10.1 (8.3, 11.8)	9.29 (7.84, 11.02)
[30–35] *N* = 141	10.2 (8.7, 14.3)	9.49 (7.99, 13.21)
[35–40] *N* = 113	10.6 (9.9, 11.8)	9.80 (9.23, 10.65)
[40–55] *N* = 80	14.3 (10.6, 18.1)	13.25 (9.82, 16.70)

*Note:* The table presents the baseline and posttreatment lower limb volumes, expressed in liters. Volumes are summarized by median and interquartile range (IQR) per BMI category.

**Table 3:** Volume reduction and log‐ratio (EC) by BMI category.

**BMI category (kg/m** ^**2**^ **)**	**Absolute volume reduction (L, median, IQR)**	**p** **value**	**Relative volume reduction (%, median, IQR)**	**p** **value**	**Effect of cure (EC) (log-ratio, median, IQR)**
[18–25] *N* = 101	0.59 [0.39–0.89]	**< 0.001**	6.7 [5.0–9.7]	**< 0.001**	−0.07 (−0.10, −0.05)
[25–30] *N* = 116	0.78 [0.50–1.16]	**< 0.001**	7.4 [5.5–9.9]	**< 0.001**	−0.08 (−0.10, −0.06)
[30–35] *N* = 141	0.83 [0.51–1.17]	**< 0.001**	7.9 [5.4–9.7]	**< 0.001**	−0.08 (−0.10, −0.06)
[35–40] *N* = 113	0.77 [0.50–1.17]	**< 0.001**	7.5 [5.0–10.4]	**< 0.001**	−0.08 (−0.11, −0.05)
[40–55] *N* = 80	1.15 [0.65–1.76]	**< 0.001**	7.5 [6.1–11.6]	**< 0.001**	−0.08 (−0.12, −0.06)

*Note:* The table summarizes treatment efficacy using absolute (in liters) and relative volume reductions (in %) and the log‐ratio (effect of cure, EC) for each BMI category. Values in bold indicate statistically significant results (*p* < 0.05).

**Table 4:** Lymph‐ICF‐LL total scores at baseline and posttreatment.

**BMI category (kg/m** ^**2**^ **)**	**Baseline Lymph-ICF-LL total scores (BT) (median, IQR)**	**Posttreatment Lymph-ICF-LL total scores (AT) (median, IQR)**	**Absolute total scores reduction (** **B** **T** − **A** **T** **) (median, IQR)**	**p** **value**	**Relative total scores reduction (%, median, IQR)**	**p** **value**
[18–25] *N* = 101	35 (15, 49)	26 (9, 39)	6.43 (3.85, 9.6)	**< 0.001**	20.0 (12.43, 38.46)	**< 0.001**
[25–30] *N* = 116	34 (22, 50)	27 (16, 38)	7.09 (4.54, 11.8)	**< 0.001**	23.7 (15.87, 31.29)	**< 0.001**
[30–35] *N* = 141	48 (33, 61)	38 (23, 52)	7.62 (5.39, 11.6)	**< 0.001**	17.28 (11.55, 27.34)	**< 0.001**
[35–40] *N* = 113	41 (23, 57)	33 (17, 46)	7.08 (4.23, 11.54)	**< 0.001**	20.69 (15.21, 29.21)	**< 0.001**
[40–55] *N* = 80	54 (42, 62)	41 (31, 52)	7.5 (5.3, 10.45)	**< 0.001**	15.97 (9.72, 21.46)	**< 0.001**

*Note:* The table summarizes functional outcomes with initial (BT) and final (AT) Lymph‐ICF‐LL total scores and absolute and relative reductions (in %) by BMI group (median, IQR). Values in bold indicate statistically significant results (*p* < 0.05).

**Table 5:** Symptom‐based questionnaires (Q1, Q6, Q7, Q27, and TOT) at baseline and posttreatment.

**BMI category (kg/m** ^**2**^ **)**	**Q1 BT (median, IQR)**	**Q1 AT (median, IQR)**	**Q6 BT (median, IQR)**	**Q6 AT (median, IQR)**	**Q7 BT (median, IQR)**	**Q7 AT (median, IQR)**	**Q27 BT (median, IQR)**	**Q27 AT (median, IQR)**
[18–25] *N* = 101	4.00 (2.00, 6.00)	2.00 (0.00, 4.00)	6.00 (4.00, 8.00)	3.00 (1.00, 5.00)	3.00 (0.00, 7.00)	2.00 (0.00, 6.00)	6.00 (3.00, 8.00)	5.00 (2.00, 7.00)
[25–30] *N* = 116	3.00 (0.75, 6.00)	2.00 (0.00, 4.00)	7.00 (4.00, 8.00)	3.00 (1.00, 4.25)	3.00 (0.00, 6.00)	2.00 (0.00, 5.00)	5.00 (2.00, 8.00)	4.00 (1.80, 6.00)
[30–35] *N* = 141	6.00 (3.00, 8.00)	3.00 (1.00, 5.00)	7.00 (5.00, 8.00)	4.00 (2.00, 5.00)	5.00 (1.00, 8.00)	4.00 (0.00, 7.00)	7.00 (4.00, 9.00)	5.00 (2.00, 8.00)
[35–40] *N* = 113	5.00 (2.00, 7.00)	2.00 (1.00, 4.00)	6.00 (4.00, 8.00)	3.00 (1.00, 4.00)	4.00 (0.00, 8.00)	2.00 (0.00, 6.00)	5.00 (1.00, 8.00)	5.00 (0.00, 7.00)
[40–55] *N* = 80	6.00 (3.00, 8.00)	3.00 (1.00, 4.25)	7.00 (6.00, 9.00)	4.00 (3.00, 6.25)	5.00 (0.00, 8.00)	4.00 (0.00, 7.00)	8.00 (5.80, 10.00)	7.00 (5.00, 9.30)

*Note:* The table summarizes scores of symptom‐related items: pain (Q1), heaviness (Q6), self‐confidence (Q7), and difficulty wearing desired clothing (Q27) at baseline (BT) and posttreatment (AT) across BMI groups.

### 3.1. Effect of CDT on Limb Volume According to the BMI

CDT led to statistically significant reductions in both absolute and relative limb volumes across all BMI categories (*p* < 0.001; Table 3). The median relative volume reduction ranged from 6.7% to 7.9% among the BMI groups.

Linear regression analysis (Table 6) demonstrated that BMI influenced the effectiveness of CDT on limb volume reduction. Compared to the reference group (18–25 kg/m^2^), only the highest BMI category (40–55 kg/m^2^) showed a significantly smaller relative volume reduction (*β* = 0.021; 95% CI: 0.003–0.039; *p* = 0.021). This suggests that, after adjustment for other factors, CDT was slightly less effective in patients with severe obesity.

**Table 6:** Linear regression of CDT effectiveness on limb volume reduction.

	**β**	**95% CI**	**p** **value**
**BMI category (kg/m** ^**2**^ **)**			
[18, 25]	—	—	—
[25, 30]	0.006	(−0.008, 0.019)	0.4
[30, 35]	0.008	(−0.006, 0.021)	0.3
[35, 40]	0.010	(−0.006, 0.025)	0.2
[40, 55]	0.021	(0.003, 0.039)	**0.021**

*Note:* The table summarizes the results of a linear regression model evaluating the effect of complex decongestive therapy (CDT) on limb volume reduction. The outcome variable was the logarithmic ratio of posttreatment to baseline limb volume. Regression coefficients (*β*), 95% confidence intervals (CIs), and *p* values are reported for each BMI category. Values in bold indicate statistically significant results (*p* < 0.05).

Figure 2 illustrates the relationship between baseline limb volume, BMI category, and the log‐transformed volume ratio (post/pretreatment). The downward trend supports the association between larger baseline volumes and greater relative reductions, while also highlighting increased variability in outcomes at higher BMI levels.

Figure 2Relationship between the initial limb volume, BMI, and posttreatment volume ratio (final volume/initial volume) in CDT. The colors represent BMI categories. (a) Initial limb volume versus volume ratio. A negative trend indicates that patients with larger initial limb volumes tend to show greater absolute reductions. However, high variability is observed, and higher BMI patients (yellow/green) generally have larger initial limb volumes. (b) BMI versus volume ratio. A slight downward trend suggests that higher BMI patients may have lower posttreatment volume ratios, but the wide distribution suggests high variability in treatment responses.

**Figure:**
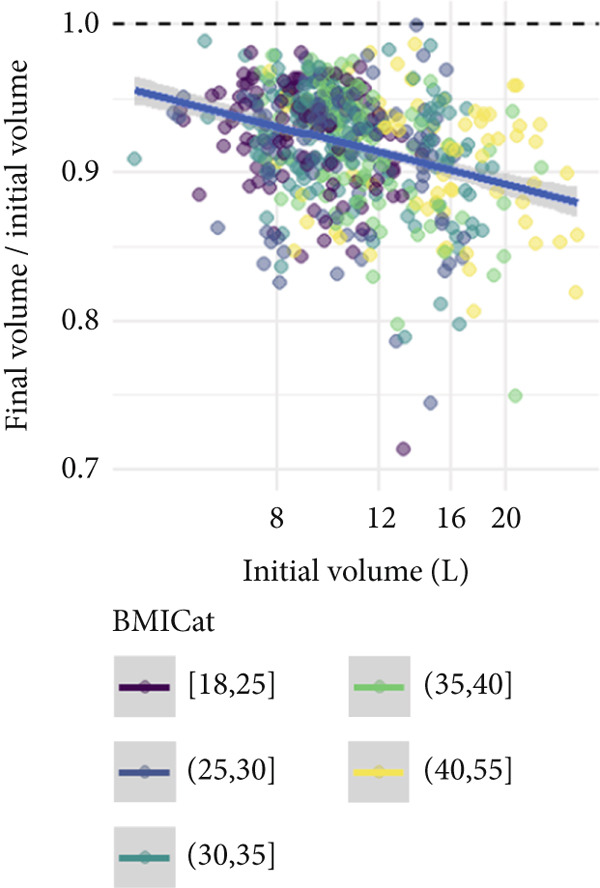
(a)

**Figure:**
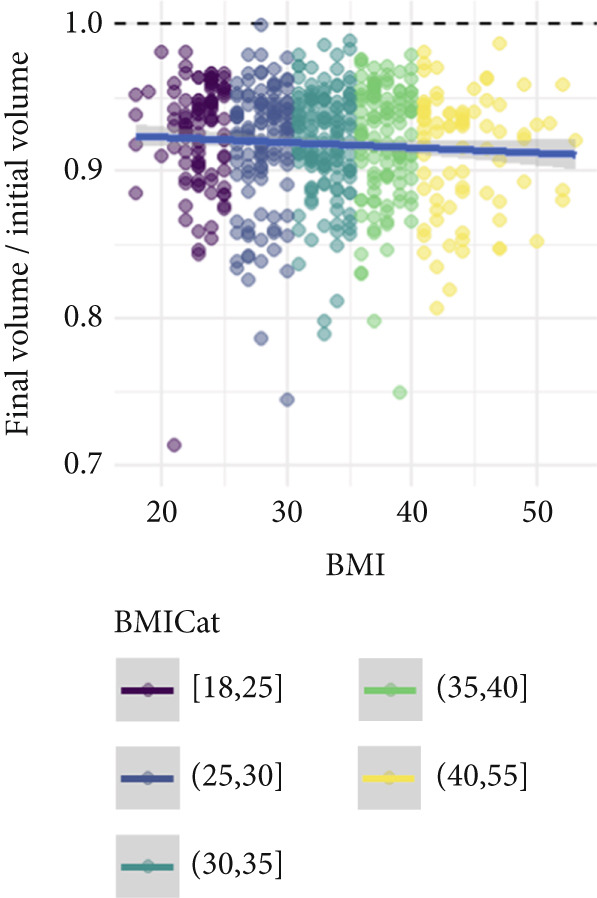
(b)

Additional covariates included in the model also influenced treatment outcomes. Unilateral lymphedema was associated with a slightly greater volume reduction (*β* = −0.100; *p* < 0.001) compared to bilateral cases (*β* = −0.092; *p* < 0.001). Age had a modest negative impact, with a 0.9% decrease in effectiveness per decade (*β* = −0.009; *p* < 0.001). The first CDT sessions were slightly more effective than follow‐up sessions, showing an additional 3.1% reduction in limb volume (*β* = −0.031; *p* < 0.001). In contrast, lymphedema etiology (primary vs. secondary) did not significantly influence treatment response (*β* = −0.009; *p* = 0.29).

### 3.2. Effect of CDT on Functional Outcomes According to the BMI

As shown in Table 4, baseline Lymph‐ICF‐LL total scores increased with rising BMI, suggesting that individuals with higher BMI experienced greater functional impairment. Following treatment, statistically significant improvements were observed across all BMI categories (*p* < 0.001 for all groups). The median absolute score reductions ranged from 6.43 to 7.62, while relative reductions varied between 15.97% and 23.7%.

Further analysis presented in Table 7, which adjusted for baseline scores using regression models, revealed no significant differences in relative improvement between BMI groups. When compared to the reference category (BMI 18–25 kg/m^2^), the *β* coefficients for other BMI groups ranged from −0.01 to +0.01, with all *p* values exceeding 0.5, indicating no statistically meaningful variation between groups.

**Table 7:** Linear regression analysis of BMI categories on Lymph‐ICF‐LL total score following CDT.

	**β**	**95% CI**	**p** **value**
**Total Lymph-ICF-LL score**			
[25, 30]	−0.01	−0.04, 0.02	0.5
[30, 35]	0.01	−0.02, 0.04	0.5
[35, 40]	0.00	−0.04, 0.03	0.8
[40, 55]	0.01	−0.03, 0.05	0.7

*Note:* The table summarizes the results of linear regression models assessing the effect of BMI categories on total Lymph‐ICF‐LL scores after complex decongestive therapy (CDT). Regression coefficients (*β*), 95% confidence intervals (CIs), and *p* values are provided for each BMI group, with the [18–25] kg/m^2^ group as reference.

In the multivariable regression models, certain factors emerged as significant predictors of treatment response. Older age was associated with slightly reduced improvement per session (*β* = 0.02, 95% CI: 0.01–0.03, *p* < 0.001). Additionally, primary lymphedema was associated with a smaller reduction in score compared to secondary forms (*β* = 0.05, *p* < 0.001). However, the session rank (initial vs. follow‐up) did not significantly influence outcomes (*β* = 0.00; *p* = 0.6).

#### 3.2.1. Item‐Level Analysis (Q1, Q6, Q7, and Q27)

Table 5 presents the results of four symptom‐specific questionnaire items. Pain (Q1) and heaviness (Q6) scores decreased significantly across all BMI groups. According to linear regression (Table 8), pain improvement was greatest in the 35–40 kg/m^2^ group (*β* = −0.11; *p* < 0.001), while the effect was smaller and not significant in the highest BMI category (*β* = −0.05; *p* = 0.12). Heaviness (Q6) showed a progressively smaller improvement with increasing BMI, with significantly reduced treatment effects in the 40–55 group (*β* = 0.11; *p* = 0.002). Self‐confidence (Q7) improved notably in the 25–30 group (*β* = −0.08; *p* = 0.002). Although *p* values for clothing‐related difficulties (Q27) were below 0.05 in the 30–35 and 35–40 groups, the confidence intervals included zero, indicating weak or uncertain evidence of a true effect.

**Table 8:** Linear regression analysis of BMI categories on item‐level functional outcomes following CDT.

**Items**	**Pain (Q1)**		**Heaviness (Q6)**		**Self-confidence (Q7)**		**Difficulty wearing desired clothing (Q27)**	
**BMI category**	**β** **(95% CI)**	**p** **value**	**β** **(95% CI)**	**p** **value**	**β** **(95% CI)**	**p** **value**	**β** **(95% CI)**	**p** **value**
[25, 30]	0.00 (−0.05, 0.06)	> 0.9	0.02 (−0.03, 0.07)	0.5	−0.08 (−0.13, −0.03)	**0.002**	0.00 (−0.04, 0.03)	0.8
[30, 35]	−0.01 (−0.06, 0.05)	0.8	0.06 (0.00, 0.11)	**0.050**	0.02 (−0.03, 0.08)	0.3	0.04 (0.00, 0.08)	**0.045**
[35, 40]	−0.11 (−0.17, −0.05)	**< 0.001**	0.07 (0.01, 0.13)	**0.028**	−0.03 (−0.08, 0.03)	0.3	0.04 (0.00, 0.08)	**0.049**
[40, 55]	−0.05 (−0.12, 0.01)	0.12	0.11 (0.04, 0.18)	**0.002**	0.01 (−0.05, 0.08)	0.7	0.03 (−0.02, 0.08)	0.2

*Note:* The table presents the results of linear regression models examining the association between BMI categories and four specific functional outcomes after CDT: pain (Q1), heaviness (Q6), self‐confidence (Q7), and difficulty wearing desired clothing (Q27). For each item and BMI group, regression coefficients (*β*), 95% confidence intervals (CIs), and *p* values are reported. Values in bold indicate statistically significant results (*p* < 0.05).

## 4. Discussion

This study is aimed at assessing the influence of BMI on the outcomes of CDT in the treatment of LL lymphedema, with a focus on limb volume reduction and improvements in functional outcomes.

Obesity is a well‐established risk factor for lymphedema, particularly in postsurgical or cancer‐related cases [42, 43]. Although this study did not examine incidence, the high proportion of overweight and obese individuals in the sample (71% with BMI > 25 kg/m^2^) aligns with these previous findings.

Patients with higher BMI presented with larger limb volumes at baseline. These findings are consistent with those of Vignes et al. [34], who observed a direct relationship between BMI and limb volume, even in nonedematous limbs. The likely explanation is that adipose tissue expands the interstitial space, promoting fluid accumulation and volume overload. Furthermore, higher BMI was associated with greater functional limitations.

Obesity may also contribute to diagnostic delays, leading to more severe presentations at the time of treatment. This likely explains why patients with higher BMI had greater absolute improvements, reflecting their higher baseline scores rather than better response to treatment.

CDT proved effective in reducing limb volume across all BMI categories. A statistically significant, though modest, reduction in treatment effect was observed only in the highest BMI group (40–55 kg/m^2^). Baseline limb volume emerged as the strongest predictor of volume reduction, consistent with the findings of Moffatt et al. [44], likely reflecting the greater mobilizable fluid load in more voluminous limbs. After adjusting for baseline volume, BMI was no longer associated with treatment response, suggesting that BMI was not an independent predictor of treatment effectiveness. These results are in line with prior studies by Zasadzka et al. [35] and Stanisic et al. [45], which also support the effectiveness of CDT regardless of body weight. This reinforces the applicability of CDT in populations with elevated BMI.

Volume reductions observed in this study were more modest than those reported for the upper limb (e.g., 36%–38% Vignes et al. [34]). Several anatomical and biomechanical factors may explain this discrepancy. The LLs are weight‐bearing, subject to higher hydrostatic pressure, and more prone to chronic changes such as fibrosis. Unlike the upper limbs, the legs cannot easily be elevated for prolonged periods, which reduces the benefit of gravity‐assisted drainage.

These considerations highlight the need to assess treatment response in relation to multiple patient‐specific factors, such as baseline volume, laterality, and chronicity, rather than BMI alone. Early intervention, before bilateral or fibrotic changes occur, remains essential.

Considering functional outcomes, patients with higher BMI had higher baseline Lymph‐ICF‐LL scores, reflecting more severe impairments. This aligns with Vieira et al. [46], who reported lower quality of life in overweight and obese individuals. While these patients showed greater absolute improvements, adjusted analyses revealed no significant difference in treatment response across BMI categories. Physical symptoms such as pain and heaviness improved across all BMI groups, with slightly smaller reductions in the highest BMI categories. Improvements in self‐confidence and clothing‐related challenges were observed consistently across all groups, indicating that some psychosocial benefits of CDT are preserved regardless of BMI. These findings suggest that excess weight may limit perceived functional benefits, particularly in the physical dimension. This highlights the value of a multidimensional approach that combines CDT with individualized support for lifestyle and weight management.

In summary, while higher BMI was associated with greater initial impairment and larger absolute gains, it was not an independent predictor of functional improvement when baseline severity was considered.

Regarding etiology, patients with primary lymphedema showed slightly smaller functional improvements compared to those with secondary forms. This is consistent with findings by Abakay et al. [47], who reported that individuals with primary lymphedema had lower baseline quality‐of‐life scores and continued to experience poorer outcomes after treatment. These differences may reflect more widespread or congenital lymphatic dysfunction, which may contribute to a reduced responsiveness to CDT. The high incidence of primary lymphedema in our study is probably due to the fact that with the help of lymphoscintigraphy, younger patients are more frequently diagnosed as having a primary lymphedema. It also reflects the referral pattern of our specialized reference center treating a 4 million inhabitant area. Also, cancer therapies are less aggressive than before explaining a possible lower incidence of secondary lymphedema.

Age was also identified as a significant influencing factor. Unlike Liao et al. [48], who found no relationship between age and initial limb volume, we observed a decrease in initial volume with increasing age. This may be explained by the tendency for BMI to decline in older adults [49], largely due to muscle mass reduction [50]. Younger patients demonstrated slightly greater improvements, likely due to better physical capacity and exercise tolerance. While Kendrová et al. [51] suggested that age is not a strong predictor of quality‐of‐life improvements in LL lymphedema, our findings imply that advancing age may indirectly hinder treatment outcomes due to the increasing difficulty of daily activities.

### 4.1. Clinical Implications

These findings not only confirm the effectiveness of CDT across BMI groups but also underline the complex interaction between obesity, baseline severity, and treatment response. In line with work by Shaw et al. [33], who demonstrated improved upper limb outcomes following weight loss, further research is needed to explore the impact of weight reduction in LL lymphedema. Integrating weight management into long‐term care could help to optimize both physical results and functional outcomes through personalized care strategies.

### 4.2. Limitations and Strengths

This study has several limitations. Although all eligible patients treated at our center during the study period were initially considered, only those with complete data and full adherence to the CDT protocol were included in the final analysis. While this approach reflects routine clinical practice, it may slightly overrepresent individuals with better engagement in care. In addition, although regression models were used to adjust for potential confounders, residual confounding cannot be completely ruled out.

Furthermore, limb volume was analyzed as a total LL measure rather than by anatomical segments (thigh, leg, and foot). Segmental data were not systematically stored, and retrospective reconstruction was not feasible without introducing bias. However, total limb volume remains the most clinically relevant and commonly reported outcome in CDT research, as it reflects the overall therapeutic effect.

The absence of formal lymphedema staging (e.g., ISL classification) limits the ability to stratify outcomes by disease severity, although baseline limb volume and functional impairment serve as reasonable clinical proxies. BMI was analyzed in categories, which may oversimplify its effects and obscure more complex associations. Finally, while data were collected in a specialized center following standardized procedures, the single‐center design may limit generalizability to broader clinical settings.

Despite these limitations, the study has notable strengths. It included a large and diverse sample of patients with varying BMI, ages, and both primary and secondary lymphedema. The combination of objective (volume) and subjective (Lymph‐ICF‐LL) outcomes provides a comprehensive assessment of treatment effects. The prospective design and standardized assessments enhance the internal validity of the findings.

Future studies should aim to validate these results in multicenter cohorts and explore additional modifiable factors—such as weight change, disease duration, and psychosocial determinants—to further personalize lymphedema care. In addition, future research should include segmental volume assessments to better characterize localized treatment responses within the limb.

## 5. Conclusion

Although a higher BMI is associated with greater initial limb volume and more severe functional impairments, it does not independently reduce the relative effectiveness of CDT when baseline differences are taken into account. CDT proved effective across all BMI categories, with absolute improvements being more pronounced in individuals with higher BMI due to more severe starting conditions.

Key predictors of treatment response included baseline limb volume, lymphedema laterality, and age. Patients with unilateral lymphedema experienced greater proportional volume reductions compared to those with bilateral involvement, and older patients showed larger relative volume decreases. In contrast, the etiology of lymphedema (primary vs. secondary) did not significantly influence treatment effectiveness.

These findings reinforce CDT as a cornerstone therapy for LL lymphedema, regardless of body weight. The results support a patient‐centered approach that considers individual clinical characteristics beyond BMI. Future research should further investigate the potential role of weight loss in optimizing CDT outcomes and improving long‐term lymphedema management.

## Conflicts of Interest

The authors declare no conflicts of interest.

## Author Contributions

**Marie Warnier**: conceptualization (supporting), data curation, and writing—original draft; **Nina Antoniolli**: conceptualization (supporting), data curation, and writing—original draft; **Benoit Bihin**: formal analysis, methodology, visualization, and writing—review and editing; **Jacqueline Frippiat**: investigation and writing—review and editing; **Chloé Meseeuw**: investigation and writing—review and editing; **Alexis Lheureux**: investigation and writing—review and editing; **Thierry Deltombe**: supervision and writing—review and editing (lead).

## Funding

No funding was received for this manuscript.

## Supporting information


**Supporting Information** Additional supporting information can be found online in the Supporting Information section. The supporting information provides the validated French version of the Lymph‐ICF‐LL questionnaire. This material is intended for reference and use by clinicians and researchers working with French‐speaking populations affected by lymphoedema.

## Data Availability

The datasets generated and analyzed during the current study are available from the corresponding author on reasonable request.
