# Supplementary material for: Impact of Body Mass Index on the Outcomes of Intensive Complex Decongestive Therapy for Lower Limb Lymphedema
Source: Int J Vasc Med. 2026 Jan 27;2026:8951146. doi: 10.1155/ijvm/8951146 (PMC12836292; doi:10.1155/ijvm/8951146)
Supplement: Supplementary file 1 — Supporting Information Additional supporting information can be found online in the Supporting Information section. The supporting information provides the validated French version of the Lymph‐ICF‐LL questionnaire. This material is intended for reference and use by clinicians and researchers working with French‐speaking populations affected by lymphoedema. [file IJVM-2026-8951146-s001.pdf]

Patient kiné vasculaire – convention lymphœdème CHU UCL Namur site Godinne – MI

SEMAINE DU: ……………………………………….

Coordonnées patient

NOM : ……………………………. Prénom :………………………………. DN……………………

CENTRE DE REFERENCE INAMI DE LA PRISE EN CHARGE DU LYMPHOEDEME

MEMBRES INFERIEURS

1) **Questionnaire lymph ICF – Lower limb**

Début score : ……….. /280
score de fin : ………. /280

Le lundi, j'entoure **Le vendredi, je fais une croix**

Le lundi : sélectionner vos réponses en entourant celles-ci : I------I------I------I------I------I------

Le vendredi : sélectionner vos réponses avec une croix

1. Système locomoteur

Avez-vous au niveau de votre membre inférieur : Pas du tout ... Vraiment beaucoup

0 1 2 3 4 5 6 7 8 9 10

1. de la douleur ? ---☺I------I------I------I------I------I------I------I------I------I------I☹

0 1 2 3 4 5 6 7 8 9 10

2. des sensations de tension au niveau de la peau ?--------☺I------I------I------I------I------I------I------I------I------I------I☹

0 1 2 3 4 5 6 7 8 9 10

3. des picotements ? --☺I------I------I------I------I------I------I------I------I------I------I☹

0 1 2 3 4 5 6 7 8 9 10

4. une infection (pour l'instant ou parfois) ------------------☺I------I------I------I------I------I------I------I------I------I------I☹

Votre membre inférieur vous semble : Pas du tout → Vraiment beaucoup

0 1 2 3 4 5 6 7 8 9 10

5. rigide ? ☺ |------|------|------|------|------|------|------|------|------|------| ☹

0 1 2 3 4 5 6 7 8 9 10

6. lourd (fatiguant) ? ☺ |------|------|------|------|------|------|------|------|------|------| ☹

2. ***Bien-être***

A cause de votre lymphoedème : Pas du tout → Vraiment beaucoup

0 1 2 3 4 5 6 7 8 9 10

7. avez-vous un manque de confiance en vous ? ☺ |------|------|------|------|------|------|------|------|------|------| ☹

A cause de votre lymphœdème, vous sentez-vous : Pas du tout → Vraiment beaucoup

0 1 2 3 4 5 6 7 8 9 10

8. triste ? ☺ |------|------|------|------|------|------|------|------|------|------| ☹

0 1 2 3 4 5 6 7 8 9 10

9. pas attirant(e) ? ☺ |------|------|------|------|------|------|------|------|------|------| ☹

0 1 2 3 4 5 6 7 8 9 10

10. frustré(e), stressé(e) ? ☺ |------|------|------|------|------|------|------|------|------|------| ☹

0 1 2 3 4 5 6 7 8 9 10

11. inquiet(e) pour votre futur ? (ex : professionnel) ☺ |------|------|------|------|------|------|------|------|------|------| ☹

0 1 2 3 4 5 6 7 8 9 10

12. désabusé(e) par rapport aux soins de santé ? (ex : manque d'information) ☺ |-----|-----|-----|-----|-----|-----|-----|-----|-----|-----| ☹

3. ***Activités domestiques***

A cause de votre lymphœdème, êtes-vous : Pas du tout → Vraiment beaucoup

0 1 2 3 4 5 6 7 8 9 10

13. devenu plus dépendant(e) des autres ? ☺ |------|------|------|------|------|------|------|------|------|------| ☹

A cause de votre lymphœdème, avez-vous des difficultés à : Pas du tout → Vraiment beaucoup

0 1 2 3 4 5 6 7 8 9 10

14. organiser des activités (ex : réaliser des tâches, respecter des RDV) ? ☺ |------|------|------|------|------|------|------|------|------|------| ☹

0 1 2 3 4 5 6 7 8 9 10

15. accomplir des tâches ménagères ? ☺ |------|------|------|------|------|------|------|------|------|------| ☹

4. ***Activités locomotrices***

A cause de votre lymphœdème, pouvez-vous : Très bien ↓ … Pas du tout ↓

0 1 2 3 4 5 6 7 8 9 10

16. rester assis une longue période ? ------------------------☺|------|------|------|------|------|------|------|------|------|------|☹

0 1 2 3 4 5 6 7 8 9 10

17. rester debout une longue période ? ---------------------☺|------|------|------|------|------|------|------|------|------|------|☹

0 1 2 3 4 5 6 7 8 9 10

18. vous agenouiller ? --☺|------|------|------|------|------|------|------|------|------|------|☹

0 1 2 3 4 5 6 7 8 9 10

19. marcher (plus de 2km) ? ---------------------------------------☺|------|------|------|------|------|------|------|------|------|------|☹ Pas pertinent

0 1 2 3 4 5 6 7 8 9 10

20. rouler à vélo ? ---☺|------|------|------|------|------|------|------|------|------|------|☹ □

0 1 2 3 4 5 6 7 8 9 10

21. conduire une voiture ? ------------------------------------☺|------|------|------|------|------|------|------|------|------|------|☹ □

0 1 2 3 4 5 6 7 8 9 10

22. utiliser les escaliers ? (ex : monter et descendre d'un bus) -☺ |------|------|------|------|------|------|------|------|------|------|☹ □

5. ***Activités sociales***

A cause de votre lymphœdème, pouvez-vous : Très bien ↓ … Pas du tout ↓ Pas pertinent

0 1 2 3 4 5 6 7 8 9 10

23. réaliser votre travail professionnel ? -------------------------☺|------|------|------|------|------|------|------|------|------|------|☹ □

0 1 2 3 4 5 6 7 8 9 10

24. faire du sport (mon sport : ....) ?-------------------☺|------|------|------|------|------|------|------|------|------|------|☹ □

0 1 2 3 4 5 6 7 8 9 10

25. avoir des activités de loisirs (mon loisir : ....) ? -☺|------|------|------|------|------|------|------|------|------|------|☹ □

0 1 2 3 4 5 6 7 8 9 10

26. avoir des activités sociales avec des amis ? (restaurant, aller à des soirées…)☺ |-----|-----|-----|-----|-----|-----|-----|-----|-----|-----|☹ □

0 1 2 3 4 5 6 7 8 9 10

27. porter les vêtements que vous voulez ? --------------------☺|------|------|------|------|------|------|------|------|------|------|☹

0 1 2 3 4 5 6 7 8 9 10

28. partir en vacances ? ---☺|------|------|------|------|------|------|------|------|------|------|☹ □
